# Tobacco Quit Intentions and Behaviors among Cigar Smokers in the United States in Response to COVID-19

**DOI:** 10.3390/ijerph17155368

**Published:** 2020-07-25

**Authors:** Sarah D. Kowitt, Jennifer Cornacchione Ross, Kristen L. Jarman, Christine E. Kistler, Allison J. Lazard, Leah M. Ranney, Paschal Sheeran, James F. Thrasher, Adam O. Goldstein

**Affiliations:** 1 Department of Family Medicine, School of Medicine, University of North Carolina at Chapel Hill, Chapel Hill, NC 27599, USA; jkristen@email.unc.edu (K.L.J.); christine_kistler@med.unc.edu (C.E.K.); Leah_Ranney@unc.edu (L.M.R.); adam_goldstein@med.unc.edu (A.O.G.); 2 Department of Social Sciences and Health Policy, Division of Public Health Sciences, Wake Forest School of Medicine, Winston Salem, NC 27157, USA; jcornacc@wakehealth.edu; 3 Lineberger Comprehensive Cancer Center, University of North Carolina at Chapel Hill, Chapel Hill, NC 27599, USA; lazard@unc.edu (A.J.L.); psheeran@email.unc.edu (P.S.); 4 Hussman School of Journalism and Media, University of North Carolina at Chapel Hill, Chapel Hill, NC 27599, USA; 5 Department of Psychology and Neuroscience, University of North Carolina at Chapel Hill, Chapel Hill, NC 27599, USA; 6 Department of Health Promotion, Education, and Behavior, Arnold School of Public Health, University of South Carolina, Columbia, SC 29208, USA; thrasher@mailbox.sc.edu

**Keywords:** COVID-19, tobacco, cigar, risk, communication, quitting, smoking cessation

## Abstract

Combustible tobacco users appear to be at greater risk for serious complications from COVID-19. This study examined cigar smokers’ perceived risk of COVID-19, quit intentions, and behaviors during the current pandemic. We conducted an online study between 23 April 2020 to 7 May 2020, as part of an ongoing study examining perceptions of different health effects of cigars. All participants used cigars in the past 30 days (n = 777). Three-quarters of the sample (76.0%) perceived they had a higher risk of complications from COVID-19 compared to non-smokers. The majority of participants (70.8%) intended to quit in the next six months due to COVID-19, and almost half of the sample (46.5%) reported making a quit attempt since the start of the COVID-19 pandemic. Far more participants reported increasing their tobacco use since COVID-19 started (40.9%) vs. decreasing their tobacco use (17.8%). Black or African American participants, participants who reported using a quitline, and participants with higher COVID-19 risk perceptions had higher intentions to quit using tobacco due to COVID-19, and higher odds of making a quit attempt since COVID-19 started. More research is needed to understand how tobacco users are perceiving COVID-19 risks and changing their tobacco use behaviors.

## 1. Introduction

Cigarette, cigar, and other combustible tobacco users appear to be at greater risk for developing serious complications from COVID-19 [1,2,3,4]. At least four systematic reviews/meta-analyses have concluded that smoking is associated with adverse outcomes associated with COVID-19 and worse disease progression [1,2,3,4], which aligns with research showing that smokers are at greater risk for contracting and experiencing severe respiratory infections [5,6]. However, some research has suggested that there are no associations between tobacco use and severity of COVID-19 complications, or that using tobacco use may confer protective benefits, such as smokers being at lower risk for contracting COVID-19 [7,8,9,10]. While there appear to be substantial limitations to this latter work [11], more research is needed on how COVID-19 is affecting tobacco users, including how they perceive COVID-19 risks and how they are altering their intentions and behaviors in response to this new threat.

Due to the potential negative health outcomes associated with COVID-19, it is possible that many tobacco users feel more vulnerable to its health effects and are trying to quit using tobacco—which aligns with both previous research and theory on risk perception [12,13]. However, it is also possible that some tobacco users are increasing their tobacco use due to stress or anxiety associated with COVID-19, which has also been documented in previous research on stress and smoking behavior [14,15,16,17]. One study recently examined smoking behaviors due to COVID-19, reporting descriptive changes in smoking in a small, convenience sample [18]. More research is needed examining how tobacco users are perceiving and reacting to COVID-19.

The goals of this study were to examine cigar smokers’ perceived risk of COVID-19, quit intentions, and behaviors during the current pandemic. In the United States (US), cigars are one of the most commonly used tobacco products with 7.2% of adults reporting smoking cigars in the past 30 days [19]. While cigars are used less frequently than cigarettes [19], dual use of cigarettes and cigars is high. Research shows that between 30–60% of cigar smokers also smoke cigarettes [20], and over 30% of cigarette smokers smoke cigars [21]. Compared to cigarette smokers, cigar smokers tend to be younger [22], include more African Americans [22], and use cigars on fewer days per month [23,24].

We also examined how other correlates were associated with COVID-19 quit intentions and behaviors, including demographics, such as age and race, and tobacco use variables, such as other tobacco use and nicotine dependence. We did so because research has found several factors in the US to be associated with risk of acquiring COVID-19 and experiencing complications, including race/ethnicity, age, and other comorbidities. Specifically, African Americans in the US are dying of COVID-19 at greater rates than white people, and are experiencing more complications [25]. Increasing age and other co-morbid conditions, like types 2 diabetes, also elevate the risk of complications, including death [26,27].

## 2. Materials and Methods

### 2.1. Participants and Procedures

We recruited participants as part of an ongoing study examining perceptions of different health effects of cigars. Specifically, this study presented different text statements about the health effects of cigars (e.g., cigar smoking can cause lung cancer) to participants, and asked them to answer if they were aware of this health effect and how effective it was at discouraging their cigar use. This study will be used to develop effective warnings for little cigar and cigarillo packages. We expanded eligibility to include all cigar users to be more representative of the cigar smoking population. Participants were recruited and compensated through Qualtrics Services—a leader in the field of survey software, deployment, methodology, and consumer research. Qualtrics has existing panels that can be used for social science research [28,29,30], and has a survey platform for designing and analyzing online surveys. Qualtrics recruited participants for our study, administered the survey that we created, compensated them, and performed an initial cleaning of the data. To be eligible for study participation, adults had to be 18 years or older, speak English, and currently use traditional large cigars, little cigars, or cigarillos (defined as using one of those products in the past 30 days). All participants were located in the US. Qualtrics recruited a final sample of 777 participants for our study from 23 April 2020 to 7 May 2020. Each survey took participants 12 min on average to complete. The University of North Carolina at Chapel Hill Institutional Review Board approved the study (study number 20-0871).

### 2.2. Measures

#### 2.2.1. Outcomes

We had two primary outcomes: (1) quit intentions in the next 6 months due to COVID-19 (average of three items, adapted from reference [31]), and (2) whether or not participants made a quit attempt since COVID-19 started (adapted from reference [32]). To assess quit intentions, we asked participants: “How interested are you in quitting smoking in the next 6 months because of COVID-19?”; “How much do you plan to quit smoking in the next 6 months because of COVID-19?”; and “How likely are you to quit smoking in the next 6 months because of COVID-19?”. For each of these three items, response options ranged from 1 (e.g., not at all interested) to 4 (e.g., very interested). To assess quit attempts, we asked participants: “Since COVID-19 started, how many times have you stopped smoking for 1 day or longer because you were trying to quit smoking?”. For this item, participants could respond with any number between 0 and 30. We dichotomized responses as no quit attempts (for responses of 0) or at least one quit attempt (responses of 1–30). We adapted the quit intentions and quit attempts items from previously validated measures by adding “COVID-19” to the item stems. We also examined change in tobacco use since COVID-19 started (newly developed item) in bivariate models but not a multivariable model for reasons described below. For this measure, we asked participants: “Since COVID-19 started infecting people in the US, would you say that your tobacco use has…” with five response options ranging from 1 (increased a lot) to 5 (decreased a lot).

#### 2.2.2. Correlates

We examined several correlates of interest, including participant characteristics, tobacco use variables, and other COVID-19 variables. Participant characteristics included age, gender, sexual orientation, race, Hispanic or Latino ethnicity, education, income, and perceived physical and mental health status [33,34]. To assess perceived physical and mental health, we asked participants: “Would you say that in general your physical health is…” and “In general, would you say your mental health is…” with response options ranging from 1 (poor) to 5 (excellent) [33,34].

Tobacco use variables included other tobacco use [35] and nicotine dependence (using items that assess nicotine dependence for users of multiple tobacco products) [36]. To assess other tobacco use, we asked participants: “In the past 30 days, which of the following products have you used at least once?” [35]. Response options included: cigarettes, smokeless tobacco (e.g., chewing tobacco, snuff, dip, or snus), e-cigarettes, and waterpipe tobacco. Participants were allowed to select more than one response option. We included e-cigarettes in this measure since e-cigarettes are regulated as a tobacco product in the US. To assess nicotine dependence, we asked participants five questions about potential symptoms of dependence on tobacco products (e.g., “During the past 30 days, have you had a strong craving to use tobacco products of any kind?”) [36]. For all five items, participants could respond “yes” (coded as 1) or “no” (coded as 0).

Finally, the survey assessed COVID-19 variables including quitline use since COVID-19 started (newly developed item), perceived risk of complications due to COVID-19 compared to non-smokers (newly developed item), COVID-19 risk perceptions (average of three items, adapted from reference [37]), and frequency of social distancing efforts (newly developed item). To assess quitline use since COVID-19 started, we asked participants: “Since COVID-19 started, have you called the Quitline (national phone number for help to quit smoking)?” Participants could respond “yes” (coded as 1) or “no” (coded as 0). To assess perceived risk of complications due to COVID-19 compared to non-smokers, we asked participants: “Please think about if you were to become infected with the coronavirus. Compared to a non-smoker, what impact do you think that your smoking has on your risk of serious health complications, hospitalization, and death from COVID-19?”. Response options ranged from 1 (My smoking gives me a much higher risk of complications from COVID-19) to 5 (My smoking gives me a much lower risk of complications from COVID-19). To assess COVID-19 risk perceptions, we asked participants three items adapted from a validated scale (e.g., How likely is it that you will become infected with COVID-19 at some point in the future?) with four response options ranging from 1 (e.g., very unlikely) to 4 (e.g., very likely) [37]. Finally, to assess frequency of social distancing efforts, we asked participants: “Physical distancing, or social distancing, is the practice of deliberately increasing the physical space between people to avoid spreading illness. How often do you currently practice daily social distancing as a result of COVID-19?”. Response options ranged from 1 (never) to 5 (always). All COVID-19 measures are presented in Supplementary Table S1. 

### 2.3. Data Analysis

We conducted analyses in SAS V.9.3 (SAS Institute, 2011, Cary, NC, USA). We first examined bivariate associations with our two outcomes (quit intentions and quit attempts) and all correlates of interest—participant characteristics, tobacco use variables, and other COVID-19 variables. We then examined bivariate associations among our two primary outcomes and changes in tobacco use since COVID-19 started (i.e., increased use, decreased use, or unchanged use). Finally, we examined associations among all correlates and our two primary outcomes in separate multivariable models. As our first outcome—quit intentions—was continuous, we entered all participant characteristic, tobacco use, and COVID-19 correlates in a multivariable linear regression model. As our second outcome—quit attempts—was dichotomous, we entered all correlates in a multivariable logistic regression model. We included all correlates in the adjusted multivariate regression models, because we did not have any a-priori hypotheses for which correlates would be associated with our outcomes. While we planned to examine changes in tobacco use as a third outcome in a multivariable, multinomial regression model, we did not run this model due to small cell counts in some of the tobacco use response options. Results from the first model include Beta coefficients (B) and standard errors (SE). If the B is positive, the interpretation is that for every 1-unit increase in the correlate, the outcome variable will increase by the B value. Results from the second model include adjusted odds ratios (aOR) and 95% confidence intervals (CI). An aOR of more than 1 means that there is a higher odds of the outcome happening with exposure to the correlate. An aOR of less than 1 means that there is a lower odds of the outcome happening. For all analyses, we set α = 0.05 and used 2-tailed statistical tests.

## 3. Results

### 3.1. Participants

Participant characteristics and tobacco use variables are presented in Table 1. The mean age of participants was 39.9 (SD: 13.4), and the majority of the sample was White (66.2%), non-Hispanic or Latino (84.9%), and straight or heterosexual (87.4%). Everyone in the sample was a cigar user and most used other tobacco products, including cigarettes (83.8%), e-cigarettes (37.7%), smokeless tobacco (21.9%), and waterpipe tobacco (13.3%). 

COVID-19 variables are presented in Table 2. Three-quarters of the sample perceived that they had a higher risk of complications from COVID-19, compared to non-smokers (76.0%). The great majority of respondents (70.8%) reported intentions to quit smoking in the next six months due to COVID-19. Almost half reported making a quit attempt (46.5%), and 22.9% reported calling a quitline due to COVID-19. Despite these positive steps, many more participants reported increasing their tobacco use a lot or a little since COVID-19 started (40.9%), compared to those that reported decreasing their tobacco use a lot or a little (17.8%). 

### 3.2. Bivariate Associations

Bivariate associations among our two outcomes with changes in tobacco use are presented in Table 3. Interestingly, reported quit intention scores (which ranged from 1–4, with higher values indicated more interest in quitting) were higher among people who reported decreasing their tobacco use (2.9, SD: 1.0) and people who reported increasing their tobacco use (2.9, SD: 1.0), compared to people who reported keeping their tobacco use the same (2.2, SD: 1.0) (*p* < 0.001 for both comparisons). Similarly, participants who reported decreasing their tobacco use were more likely to report making a quit attempt (63.0%), and participants who reported increasing their tobacco use were also more likely to report making a quit attempt (57.6%), compared to participants who reported keeping their tobacco use the same (28.4%) (*p* < 0.001). Finally, participants who reported making a quit attempt had higher quit intentions (3.2, SD: 0.8) than participants who did not report making a quit attempt (2.1, SD: 1.0) (*p* < 0.001). All bivariate associations among correlates of interest and our two outcomes (quit intentions because of COVID-19 and quit attempts since COVID-19 started) are presented in Supplementary Tables S2 and S3.

### 3.3. Multivariate Associations

#### 3.3.1. Participant Characteristics

Black or African American participants had higher quit intentions due to COVID-19 (B = 0.25, *p* = 0.002) and had higher odds of making a quit attempt since COVID-19 started (aOR: 1.94, 95% CI: 1.26, 2.97), compared to White participants (Table 4). Better mental health status was associated with higher quit intentions due to COVID-19 (B = 0.10, *p* = 0.005).

#### 3.3.2. Tobacco Use Variables

Participants who smoked cigars and cigarettes had lower odds of making a quit attempt since COVID-19 started (aOR: 0.56, 95% CI: 0.34, 0.94), compared to participants who smoked cigars and did not smoke cigarettes. In addition, participants who smoked cigars and used smokeless tobacco had higher odds of making a quit attempt since COVID-19 started (aOR: 2.42, 95% CI: 1.47, 3.98), compared to participants who smoked cigars and did not use smokeless tobacco.

#### 3.3.3. COVID-19 Variables

Participants who reported calling a quitline had higher quit intentions due to COVID-19 (B = 0.47, *p* < 0.001), and had higher odds of making a quit attempt since COVID-19 started (aOR: 8.66, 95% CI: 4.89, 15.34), compared to participants who did not report calling a quitline. Participants who believed they had a higher risk of complications due to COVID-19, compared to non-smokers had higher quit intentions due to COVID-19 (B = 0.47, *p* < 0.001) than participants who believed they had the same risk of complications. In addition, having higher risk perceptions of COVID-19 was associated with higher quit intentions due to COVID-19 (B = 0.38, *p* < 0.001) and higher odds of making a quit attempt since COVID-19 started (aOR: 1.31, 95% CI: 1.04, 1.64). Finally, greater social distancing efforts were associated with higher quit intentions due to COVID-19 (B = 0.11, *p* = 0.01).

## 4. Discussion

The COVID-19 epidemic has negatively impacted millions of people around the globe, particularly those with co-morbid conditions that elevate risk of complications, including death [26]. Combustible tobacco use appears to be a major risk factor for worse outcomes if a tobacco user contracts COVID-19 [1,2]. Results from our study suggest that most tobacco users correctly perceive themselves to be at higher risk of COVID-19 complications, and this risk appears to relate to intentions to quit using tobacco and attempting to quit. This relationship appears even stronger for African Americans, who are at an even higher risk of complications and death due to COVID-19 [25]. Yet, despite this knowledge and these intentions / behaviors, over twice as many tobacco users reported increasing rather than decreasing their tobacco use in our study.

These results have implications for practitioners, policy-makers, and public health agencies. For practitioners, out results suggest that their patients who use tobacco have a heightened interest in quitting because of COVID-19. Each year around two thirds of tobacco users want to quit and make a quit attempt [38,39]. That the majority of tobacco users in our study, most of whom used multiple tobacco products and displayed multiple symptoms of nicotine dependence, intended to quit and made a quit attempt is important. To translate quit attempts into successful cessation, support for tobacco users should be made available during this time, including increased access to nicotine replacement therapy, virtual support with tobacco treatment counselors, and mental health assistance, particularly since better perceived mental health was associated with increased intentions to quit in our study. Tailoring support to sub-groups of tobacco users may also be important. For instance, tobacco users who have increased their tobacco use in response to COVID-19 may need additional help with higher dependency, as well as with coping strategies for stress and anxiety. Those who have decreased their tobacco use have an even greater chance of successfully quitting with clinician support.

Tobacco users with higher COVID-19 risk perceptions appear to have higher quit intentions and higher odds of making a quit attempt since COVID-19 started. However, not all smokers believed that they had a higher or similar risk of COVID-19 complications, compared to non-smokers. These findings indicate that clear and consistent messages about risks of COVID-19 to tobacco users are needed. Importantly, these messages may need to evolve as more data on tobacco use and COVID-19 become available.

Interestingly, we found that more people reported increasing their tobacco use (41%) than decreasing their tobacco use (18%). Only one previous study, to our knowledge, has examined how tobacco users have changed their behaviors in response to COVID-19 and reported similar results. In this study of 345 dual cigarette and e-cigarette users, 30.3% of participants reported increasing their cigarette use and 29.1% reported increasing their e-cigarette use since learning of COVID-19 [18]. Extending these findings, we found interesting relationships between changes in tobacco use, quit intentions, and quit attempts since COVID-19 started. Specifically, quit intentions and odds of making a quit attempt were higher in people who reported decreasing their tobacco use and people with higher COVID-19 risk perceptions, which is in line with what is often called the “vulnerability hypothesis”. Indeed, both theory and research support the idea that as individuals feel more vulnerable to the health effects of smoking, they are more likely to intend to quit smoking, make quit attempts, and successfully quit [12,13]. 

However, we also found that people who reported increasing their tobacco use also reported higher quit intentions and had higher odds of making a quit attempt—in line with research on the “stress hypothesis”. It is possible that these people want to quit, but are stressed or anxious, and are increasing their tobacco use despite their ‘good’ intentions. To this point, we did find that better mental health status was associated with higher intentions to quit. In addition, people may be bored or have stockpiled tobacco products before sheltering in place orders, which could have increased their tobacco use. Finally, it is also possible that people may want to quit but are not able to easily access evidence-based cessation resources like pharmacotherapy or behavioral support [40]. 

We also found that over a fifth of participants in our sample reported calling a quitline because of COVID-19, and that those who reported calling a quitline had higher quit intentions and attempts. Although the majority of US smokers are aware of quitlines [41,42], they only reach 1–2% of smokers nationally [43,44]. However, states have been able to increase quitline reach through targeted efforts. For instance, by adding hours of operation and implementing a cigarette tax, Maine was able to reach over 6% of smokers [45]. In addition, the national tobacco education campaign “Tips From Former Smokers (Tips),” which tagged many of its ads with 1-800-QUIT-NOW, led to 170,000 additional quitline calls over a three-month period [46]. It is possible that characteristics of our study sample—which was comprised of many heavy tobacco users—meant that participants were more likely to want to quit and use available resources to do so. It is also possible that many people answered this question favorably because of a social desirability bias. Regardless, our study findings indicate that many tobacco users reported wanting to quit because of COVID-19 and reported using a quitline for help. That many participants did not quit, suggests that tobacco quitlines may need more resources and COVID-19 specific information to help smokers quit.

Finally, we identified several characteristics that were associated with COVID-19 quit intentions and attempts. For instance, Black or African American participants had higher quit intentions and attempts due to COVID-19. African Americans in the US are dying of COVID-19 at greater rates than white people and experiencing higher rates of complications [25]. This may explain why we observed an increased desire to quit smoking among these participants. We also found that people who smoked cigarettes (in addition to cigars) had lower odds of making a quit attempt, compared to people who did not smoke cigarettes, and that people who used smokeless tobacco (in addition to smoking cigars) had higher odds of making a quit attempt, compared to people who did not use smokeless tobacco. It is possible that smokeless tobacco users had higher odds of making a quit attempt, because they do not want to put their fingers in their mouth to use these products. It is also possible that people are substituting some tobacco products with others. For instance, people could be switching to using smokeless tobacco because they are not combustible. Further research, especially longitudinal data, are needed to understand how people are changing their patterns of tobacco use in response to COVID-19. To our knowledge, we are the first to use and modify previously available measures to apply to COVID-19. As tobacco control researchers continue to collect data on tobacco use and COVID-19, the items we developed—especially those related to COVID-19 specific quit intentions, quit attempts, and perceived risk—can be used.

### Limitations

There are several limitations to this study. First, all data were self-reported, which introduces threats of social desirability bias. Second, this was a one-time cross-sectional study, which means that we were unable to assess temporality of associations or trends over time. Future longitudinal research is needed to understand how COVID-19 is changing participants’ smoking and quitting behaviors. Third, all participants were recruited online and are not representative of the US population or of tobacco users, which means that study findings may not apply to other countries or groups of tobacco users. Fourth, since this was a cross-sectional study and there was no possibility of randomizing participants to different exposures, we cannot make any claims of causality. We are careful throughout this article to use language like “correlates” rather than “predictors” and “associated with” rather than “causes.” Fifth, we created several measures in this study that have not been previously used (e.g., changes in tobacco use since COVID-19 started) given the novelty of COVID-19 and the rapid research needed to understand it. 

## 5. Conclusions

Many tobacco users included in our study perceived that they were at risk higher risk of COVID-19 complications, reported intending to quit due to COVID-19, and reported making a quit attempt since COVID-19 started, however, more tobacco users reported increasing their tobacco use due to COVID-19 than decreasing it. More research is needed to understand how tobacco users are perceiving risks of COVID-19 and changing their tobacco use behaviors. 

## Figures and Tables

**Table 1:** Participant characteristics, n = 777.

Variable	N (%)	Mean (SD)
Age	--	39.9 (13.4)
Gender		
Male	389 (50.1%)	--
Female	380 (48.9%)	--
Transgender or other	8 (1.0%)	--
Sexual orientation		
Heterosexual or straight	679 (87.4%)	
Gay, lesbian, bisexual, other	98 (12.6%)	
Ethnicity		
Not Hispanic or Latino	659 (84.9%)	--
Hispanic or Latino	117 (15.1%)	--
Race		
White	514 (66.2%)	--
Black or African American	179 (23.0%)	--
American Indian or Alaska Native	19 (2.5%)	--
Asian	33 (4.3%)	--
Pacific Islander	2 (0.3%)	--
Other	30 (3.9%)	--
Education		
High school degree or less	218 (28.1%)	--
Some college	161 (20.7%)	--
Bachelor’s or Associate’s degree	259 (33.3%)	--
Graduate degree	139 (17.9%)	--
Income		
Below $25,000 per year	192 (24.7%)	--
Between $25,000 and $49,999 per year	192 (24.7%)	--
Between $50,000 and $74,999 per year	147 (18.9%)	--
Between $75,000 and $100,000 per year	112 (14.4%)	--
Above $100,000 per year	134 (17.3%)	--
Perceived physical health ^a^	--	3.5 (1.0)
Perceived mental health ^a^	--	3.6 (1.1)
Other tobacco products used in the past 30 days ^b^		
Cigarette	651 (83.8%)	
E-cigarette	293 (37.7%)	
Smokeless tobacco	170 (21.9%)	
Waterpipe tobacco	103 (13.3%)	
Nicotine dependence ^c^		3.2 (1.5)

^a^ Scores range from 1–5, higher values indicate better perceived health. ^b^ Categories are not mutually exclusive. ^c^ Scores range from 0–5, higher values indicate more nicotine dependence.

**Table 2:** COVID-19 variables, n = 777.

Variable	N (%)	Mean (SD)
Quit intentions in next 6 months because of COVID-19 ^a^	--	2.6 (1.1)
Quit attempt since COVID-19 started		
No	416 (53.5%)	
Yes	361 (46.5%)	
Change in tobacco use since COVID-19 started		
Increased a lot or a little	318 (40.9%)	--
Stayed about the same	321 (41.3%)	--
Decreased a lot or a little	138 (17.8%)	--
Quitline use due to COVID-19		
No	599 (77.1%)	
Yes	178 (22.9%)	
Perceived risk of complications due to COVID-19, compared to non-smokers		
Much higher/slightly higher risk	590 (76.0%)	
Same risk	130 (16.8%)	
Lower or slightly lower risk	56 (7.2%)	
COVID-19 risk perceptions ^b^	--	2.6 (0.9)
Frequency of social distancing efforts ^c^		4.6 (0.8)

^a^ Scores range from 1–4, higher values indicate more interest in quitting. When dichotomized, 70.8% of participants expressed intention to quit using tobacco in the next 6 months. ^b^ Scores range from 1–4, higher values indicate higher risk perceptions. ^c^ Scores range from 1–5, higher values indicate more social distancing.

**Table 3:** Bivariate associations among changes in tobacco use since COVID-19 started, quit intentions due to COVID-19, and whether or not participants made a quit attempt since COVID-19 started, n = 777.

Change in Tobacco Use since COVID-19 Started	Quit Intentions Due to COVID-19 ^a^ Mean (SD)	*p*-Value ^b^	Quit Attempt since COVID-19 Started % of Group Who Made a Quit Attempt (n/row n)	*p*-Value ^c^
Increased a lot or a little	2.9 (1.0)	*p* < 0.001	57.6% (183/318) ^d^	*p* < 0.001
Stayed about the same	2.2 (1.0)		28.4% (91/321)	
Decreased a lot or a little	2.9 (1.0)		63.0% (87/138)	
**Quit Attempt since COVID-19 Started**				
No	2.1 (1.0)	*p* < 0.001	--	
Yes	3.2 (0.8)		--	

^a^ Scores range from 1–4, higher values indicate more interest in quitting. ^b^ *p*-value from ANOVA or t-test. ^c^ *p*-value from chi-square test. ^d^ Row percentages are provided. For instance, the first percentage can be interpreted as: 318 participants reported increasing their tobacco use a lot or a little since COVID-19 participants. Of these 318 participants, 183 made a quit attempt. So, 57.6% of participants who increased their tobacco use a lot or a little reported making a quit attempt since COVID-19 started.

**Table 4:** Multivariable associations between correlates and quit intentions due to COVID-19 and whether or not participants made a quit attempt since COVID-19 started, n = 777.

Variable	Quit Intentions Due to COVID-19 B (SE), *p*-Value	Quit Attempt since COVID-19 Started aOR (95% CI)
Age	B = −0.003 (0.003), *p* = 0.34	0.99 (0.97, 1.00)
Gender		
Male	REF	REF
Female	B = 0.06 (0.07), *p* = 0.37	1.14 (0.78, 1.69)
Transgender or other	B = 0.34 (0.34), *p* = 0.32	0.63 (0.10, 3.86)
Sexual orientation		
Heterosexual or straight	REF	REF
Gay, lesbian, bisexual, other	B = 0.005 (0.10), *p* = 0.96	1.15 (0.67, 1.98)
Ethnicity		
Not Hispanic or Latino	REF	REF
Hispanic or Latino	B = 0.02 (0.09), *p* = 0.81	1.64 (0.96, 2.78)
Race		
White	REF	REF
Black or African American	**B = 0.25 (0.08), *p* = 0.002**	**1.94 (1.26, 2.97)**
American Indian or Alaska Native	B = 0.22 (0.20), *p* = 0.27	0.63 (0.19, 2.03)
Asian	B = 0.26 (0.16), *p* = 0.11	0.99 (0.38, 2.58)
Pacific Islander	B = −0.09 (0.63), *p* = 0.89	NA (cell sizes too small)
Other	B = 0.17 (0.18), *p* = 0.35	1.18 (0.47, 2.98)
Education		
High school degree or less	REF	REF
Some college	B = −0.09 (0.09), *p* = 0.33	0.91 (0.55, 1.51)
Bachelor’s or Associate’s degree	B = 0.05 (0.09), *p* = 0.55	1.06 (0.66, 1.70)
Graduate degree	B = 0.15 (0.12), *p* = 0.21	**2.50 (1.24, 5.02)**
Income		
Below $25,000 per year	REF	REF
Between $25,000 and $49,999 per year	**B = 0.23 (0.09), *p* = 0.01**	**1.91 (1.15, 3.17)**
Between $50,000 and $74,999 per year	**B = 0.39 (0.10), *p* < 0.001**	**2.05 (1.19, 3.55)**
Between $75,000 and $100,000 per year	**B = 0.35 (0.11), *p* = 0.002**	**2.00 (1.06, 3.77)**
Above $100,000 per year	B = 0.11 (0.12), *p* = 0.38	1.61 (0.81, 3.22)
Perceived physical health	B = 0.03 (0.04), *p* = 0.43	0.94 (0.75, 1.18)
Perceived mental health	**B= 0.10 (0.04), *p* = 0.005**	0.95 (0.78, 1.16)
Cigarette user		
No	REF	REF
Yes	B = −0.007 (0.09), *p* = 0.93	**0.56 (0.34, 0.94)**
E-cigarette user		
No	REF	REF
Yes	B = 0.01(0.07), *p* = 0.89	0.98 (0.68, 1.42)
Smokeless tobacco user		
No	REF	REF
Yes	B = 0.05 (0.09), *p* = 0.57	**2.42 (1.47, 3.98)**
Waterpipe tobacco user		
No	REF	REF
Yes	B = 0.05 (0.10), *p* = 0.85	0.92 (0.52, 1.63)
Nicotine dependence	B = −0.007 (0.02), *p* = 0.75	1.11 (0.98, 1.26)
Quitline use due to COVID-19		
No	REF	REF
Yes	**B = 0.47 (0.09), *p* < 0.001**	**8.66 (4.89, 15.34)**
Perceived risk of complications due to COVID-19 compared to non-smoker		
Much higher/slightly higher risk	**B = 0.47 (0.09), *p* < 0.001**	1.52 (0.92, 2.51)
Same risk	REF	REF
Lower or slightly lower risk	B = 0.11 (0.14), *p* = 0.42	1.50 (0.68, 3.28)
COVID-19 risk perceptions	**B = 0.38 (0.04), *p* < 0.001**	**1.31 (1.04, 1.64)**
Frequency of social distancing efforts	**B = 0.11 (0.04), *p* = 0.01**	1.18 (0.92, 1.50)

Note: All variance inflation factor (VIF) scores were less than 2, indicating a low risk of multicollinearity. Boldface indicates statistical significance at *p* < 0.05.

## Supplementary Materials

The following are available online at https://www.mdpi.com/1660-4601/17/15/5368/s1, Table S1: Measures, Table S2: Bivariate associations between variables and quit intentions due to COVID-19 (higher values indicate higher intentions to quit), Table S3: Bivariate associations between variables and whether participants made a quit attempt or not since COVID-19 started.
