# Supplementary material for: Tobacco Quit Intentions and Behaviors among Cigar Smokers in the United States in Response to COVID-19
Source: Int J Environ Res Public Health. 2020 Jul 25;17(15):5368. doi: 10.3390/ijerph17155368 (PMC7432467; doi:10.3390/ijerph17155368)
Supplement: Supplementary file 1 [file ijerph-17-05368-s001.pdf]

Supplementary Tables

Table S1. Measures

**Construct**	**Item(s)**	**Response scale**
Instructions	Over the past few months, a new respiratory disease, coronavirus 2019 or COVID-19, has been in the news as it spreads around the world and within the United States.<br><br>The next few questions are about the recent coronavirus outbreak.	--
Frequency of social distancing efforts	Physical distancing, or social distancing, is the practice of deliberately increasing the physical space between people to avoid spreading illness. How often do you currently practice **daily** social distancing as a result of COVID-19?	5 = Always<br>4 = Usually<br>3 = About half the time<br>2 = Seldom<br>1 = Never
Quit attempts since COVID-19 started	Since COVID-19 started, how many times have you stopped smoking for 1 day or longer because you were trying to quit smoking?	Free response 0-30
Quitline use due to COVID-19	Since COVID-19 started, have you called the Quitline (national phone number for help to quit smoking)?	1 = Yes<br>0 = No
Quit intentions due to COVID-19	How interested are you in quitting smoking in the next 6 months because of COVID-19?	4=Very interested<br>3=Somewhat interested<br>2=A little interested<br>1=Not at all interested
	How much do you plan to quit smoking in the next 6 months because of COVID-19?	4=Very much<br>3=Somewhat<br>2=A little<br>1=Not at all
	How likely are you to quit smoking in the next 6 months because of COVID-19?	4=Very likely<br>3=Somewhat likely<br>2=A little likely<br>1=Not at all likely
Perceived risk of complications due to COVID-19 compared to non-smoker	Please think about if you were to become infected with the coronavirus.<br><br>Compared to a non-smoker, what impact do you think that your smoking has on your risk of serious health complications, hospitalization, and death from COVID-19? Compared to a non-smoker…	1 = My smoking gives me a much higher risk of complications from COVID-19<br>2 = My smoking gives me a slightly higher risk of complications from COVID-19<br>3 = My smoking gives me about the same risk of complications from COVID-19<br>4 = My smoking gives me a slightly lower risk of complications from COVID-19

		5 = My smoking gives me a much lower risk of complications from COVID-19
Change in tobacco use since COVID-19 started	Since COVID-19 started infecting people in the US, would you say that your tobacco use has…	1 = Increased a lot<br>2 = Increased a little<br>3 = Stayed about the same<br>4 = Decreased a little<br>5 = Decreased a lot
COVID-19 risk perceptions	How likely is it that you will become infected with COVID-19 at some point in the future?	4=Very Likely<br>3=Likely<br>2=Unlikely<br>1=Very Unlikely
	How fearful are you about becoming infected with COVID-19 at some point in the future?	4=Very fearful<br>3=Fearful<br>2=A little fearful<br>1=Not at all fearful
	How vulnerable do you feel to becoming infected with COVID-19?	4=Very vulnerable<br>3=Vulnerable<br>2=A little vulnerable<br>1=Not at all vulnerable

Table S2. Bivariate associations between correlates and quit intentions due to COVID-19 (higher values indicate higher intentions to quit)

Variable	Mean (SD)	p-value [a]	Correlation value	p-value [b]
Age	--	--	r = -0.14	p<0.001
Gender				
Male	2.7 (1.0)	p=0.04	--	--
Female	2.5 (1.1)		--	
Transgender or other	2.7 (0.7)		--	
Sexual orientation				
Heterosexual or straight	2.6 (1.1)	p=0.22	--	--
Gay, lesbian, bisexual, other	2.7 (1.1)		--	
Ethnicity				
Not Hispanic or Latino	2.6 (1.1)	p=0.005	--	--
Hispanic or Latino	2.9 (1.0)		--	
Race				
White	2.5 (1.1)	p=0.01	--	--
Black or African American	2.7 (1.1)		--	
American Indian or Alaska Native	2.8 (1.0)		--	
Asian	3.1 (0.8)		--	
Pacific Islander	3.3 (0.9)		--	
Other	2.5 (1.0)		--	
Education				
High school degree or less	2.4 (1.1)	p<0.001	--	--
Some college	2.4 (1.1)		--	
Bachelor's or Associate's degree	2.6 (1.0)		--	
Graduate degree	3.1 (0.9)		--	
Income				
Below $25,000 per year	2.2 (1.05)	p<0.001	--	--
Between $25,000 and $49,999 per year	2.5 (1.08)		--	
Between $50,000 and $74, 999 per year	2.7 (1.0)		--	
Between $75,000 and $100,000 per year	2.9 (1.0)		--	
Above $100,000 per year	2.9 (1.0)			
Perceived physical health	--	--	r = 0.20	p<0.001
Perceived mental health	--	--	r = 0.20	p<0.001
Cigarette user				
No	2.7 (1.0)	p=0.47	--	--
Yes	2.6 (1.1)		--	
E-cigarette user				
No	2.5 (1.1)	p=0.006	--	--
Yes	2.7 (1.0)		--	
Smokeless tobacco user				
No	2.5 (1.1)	p<0.001	--	--
Yes	3.0 (1.0)		--	
Waterpipe tobacco user				
No	2.6 (1.1)	p=0.009	--	--

Yes	2.9 (0.9)		--	
Nicotine dependence	--	--	r = 0.15	p<0.001
Quitline use due to COVID-19				
No	2.4 (1.1)	p<0.001	--	--
Yes	3.3 (0.7)		--	
Perceived risk of complications due to COVID-19, compared to non-smokers				
Much higher/slightly higher risk	2.8 (1.0)	p<0.001	--	--
Same risk	2.0 (1.0)		--	
Lower or slightly lower risk	2.3 (1.1)		--	
COVID-19 risk perceptions [c]	--	--	r = 0.46	p<0.001
Frequency of social distancing efforts	--	--	r = 0.20	p<0.001

[a] p-values are from t-tests or ANOVAs
[b] p-values are from correlation tests

Table S3. Bivariate associations between variables and whether participants made a quit attempt or not since COVID-19 started

Variable	% of group who made a quit attempt [a] (n / row n)	p-value [b]	Mean (SD) among those who made a quit attempt	Mean (SD) among those who did not make a quit attempt	p-value [c]
Age	--	--	42.5 (13.9)	36.9 (12.3)	p<0.001
Gender					
Male	51.7% (201 / 389)	p=0.01	--	--	--
Female	41.3% (157 / 380)		--	--	
Transgender or other	37.5% (3 / 8)		--	--	
Sexual orientation					
Heterosexual or straight	45.5% (309 / 679)	p=0.16	--	--	--
Gay, lesbian, bisexual, other	53.1% (52 / 98)		--	--	
Ethnicity					
Not Hispanic or Latino	43.7% (288 / 659)	p<0.001	--	--	
Hispanic or Latino	61.5% (72 / 117)		--	--	
Race					
White	43.2% (222 / 514)	p=0.05	--	--	--
Black or African American	53.6% (96 / 179)		--	--	
American Indian or Alaska Native	42.11% (8 / 19)		--	--	
Asian	60.6% (20 / 33)		--	--	
Pacific Islander	100.0% (2 / 2)		--	--	
Other	43.3% (13 / 30)		--	--	
Education					
High school degree or less	37.2% (81 / 281)	p<0.001	--	--	--
Some college	36.7% (59 / 161)		--	--	
Bachelor's or Associate's degree	44.8% (116 / 259)		--	--	
Graduate degree	75.5% 105 / 139		--	--	
Income					
Below $25,000 per year	31.8% (61 / 192)	p<0.001	--	--	--
Between $25,000 and $49,999 per year	42.7% (82 / 192)		--	--	
Between $50,000 and $74, 999 per year	48.3% (71 /147)		--	--	
Between $75,000 and $100,000 per year	56.3% (63 / 112)		--	--	
Above $100,000 per year	62.7% (84 / 134)		--	--	
Perceived physical health	--	--	3.7 (1.0)	3.4 (1.0)	p<0.001
Perceived mental health	--		3.7 (1.1)	3.5 (1.1)	p=0.004
Cigarette user					
No	57.1% (72 / 126)	p=0.009	--	--	--

Yes	44.4% (289 / 651)		--	--	
E-cigarette user					
No	42.8% (207 / 484)	p=0.008	--	--	--
Yes	52.6% (154 / 293)		--	--	
Smokeless tobacco user					
No	38.4% (233 / 607)	p<0.001	--	--	--
Yes	75.3% (128 / 170)		--	--	
Waterpipe tobacco user					
No	43.3% (292 / 674)	p<0.001	--	--	--
Yes	67.0% (69 / 103)		--	--	
Nicotine dependence	--	--	3.5 (1.4)	2.9 (1.6)	p<0.001
Quitline use due to COVID-19					
No	33.9% (203 / 599)	p<0.001	--	--	--
Yes	88.8% (158 / 178)		--	--	
Perceived risk of complications due to COVID-19 compared to non-smokers					
Much higher/slightly higher risk	49.2% (290 / 590)	p=0.004	--	--	--
Same risk	33.1% (43 / 130)		--	--	
Lower or slightly lower risk	48.21% (27 / 56)		--	--	
COVID-19 risk perceptions [c]	--	--	2.8 (0.8)	2.3 (0.8)	p<0.001
Frequency of social distancing efforts	--		4.6 (0.7)	4.5 (9.9)	p=0.10

[a] Row percentages are provided. For instance, in the first cell, 57.14% of smokers made a quit attempt and 44.39% of non-smokers made a quit attempt.
[b] p-values are from chi-square tests
[c] p-values are from t-tests
